# The topography of a continental indenter: The interplay between crustal deformation, erosion, and base level changes in the eastern Southern Alps

**DOI:** 10.1002/2016JF003884

**Published:** 2017-01-24

**Authors:** J. Robl, B. Heberer, G. Prasicek, F. Neubauer, S. Hergarten

**Affiliations:** ^1^ Department of Geography and GeologyUniversity of SalzburgSalzburgAustria; ^2^ Institute of Earth and Environmental Sciences‐GeologyUniversity of FreiburgFreiburg im BreisgauGermany

**Keywords:** indenter tectonics, European Southern Alps, landscape evolution, channel metric, drainage divide migration, base level change

## Abstract

The topography of the eastern Southern Alps (ESA) reflects indenter tectonics causing crustal shortening, surface uplift, and erosional response. Fluvial drainages were perturbed by Pleistocene glaciations that locally excavated alpine valleys. The Late Miocene desiccation of the Mediterranean Sea and the uplift of the northern Molasse Basin led to significant base level changes in the far field of the ESA and the Eastern Alps (EA), respectively. Among this multitude of mechanisms, the processes that dominate the current topographic evolution of the ESA and the ESA‐EA drainage divide have not been identified. We demonstrate the expected topographic effects of each mechanism in a one‐dimensional model and compare them with observed channel metrics. We find that the normalized steepness index increases with uplift rate and declines from the indenter tip in the northwest to the foreland basin in the southeast. The number and amplitude of knickpoints and the distortion in longitudinal channel profiles similarly decrease toward the east. Changes in slope of *χ*‐transformed channel profiles coincide spatially with the Valsugana‐Fella fault linking crustal stacking and uplift induced by indenter tectonics with topographic evolution. Gradients in *χ* across the ESA‐EA drainage divide imply an ongoing, north directed shift of the Danube‐ESA watershed that is most likely driven by a base level rise in the northern Molasse basin. We conclude that the regional uplift pattern controls the geometry of ESA‐EA channels, while base level changes in the far field control the overall architecture of the orogen by drainage divide migration.

## Key Points

- Channels of the eastern Southern Alps reveal information on tectonics and base level changes
- A NW‐SE decrease in normalized steepness index coincides with decreasing uplift rates and level of exhumation
- Base level changes in the far field control the north directed migration of major watershed

## 1 Introduction

Crustal deformation, climatic regime, and base level changes in the far field control the overall architecture of alpine drainage systems including the course of rivers, channel gradients, and the position of drainage divides [*Miller and Slingerland*, 2006; *Willett et al*., 2006; *Stüwe et al*., 2008; *Bonnet*, 2009; *Champagnac et al*., 2012; *Willett et al*., 2014; *Yang et al*., 2015]. Rivers follow topographic gradients that evolve during continental collision and in turn incise into bedrock and shape valleys [e.g., *Robl et al*., 2008b]. In principle, fluvial incision in concert with erosional hillslope processes tend to establish a topographic steady state where uplift rates are balanced by erosion rates [e.g., *Montgomery*, 2001]. However, modeling studies imply that progressive shortening and deformation displace streams horizontally and vertically, deform drainage basins [e.g., *Hallet and Molnar*, 2001], impose flow directions upon large rivers by the activation of crustal scale strike‐slip faults (e.g., orogen‐parallel valleys), and thus distort the orogen‐wide drainage network [*Robl et al*., 2008a, 2008b]. Drainages respond to such perturbations via adjustment of watersheds by divide migration and river piracy events [*Stüwe et al*., 2008; *Castelltort et al*., 2012; *Willett et al*., 2014; *Goren et al*., 2015; *Yang et al*., 2015] and via adaption of channel gradients until they are in line with uplift rate and discharge of the river. In general, there is a time lag between the perturbation of the drainage system (tectonic or climatic) and the adjustment of the landscape by erosional surface processes. Hence, most mountain ranges are characterized by a transient topographic state featuring numerous mobile knickpoints and paleo‐surfaces [e.g., *Schlunegger and Schneider*, 2005; *Norton et al*., 2008; *Hergarten et al*., 2010; *Norton et al*., 2010].

**Figure 1:**
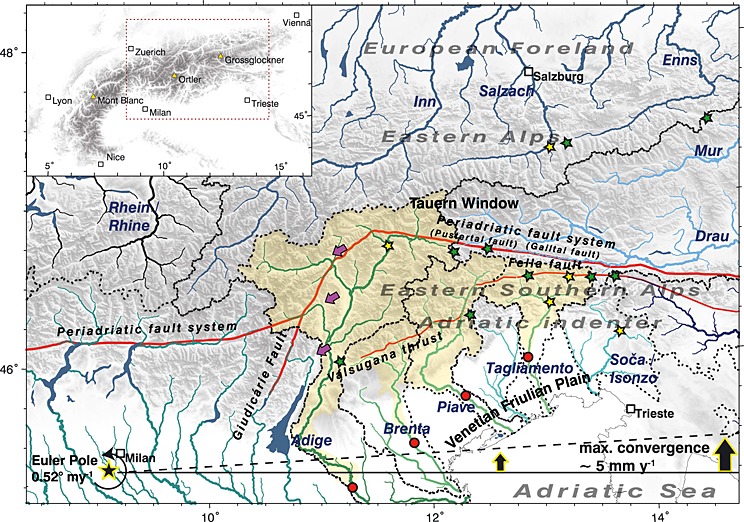
Topographic map and geodynamic setting of the study area and adjacent alpine domains. Drainage systems are color coded according to their base levels. Green, blue, and black colors indicate rivers draining toward the Mediterranean Sea, the Black Sea, and the Atlantic Ocean, respectively. Drainage divides are marked by black dotted lines. Morphometric analyses are performed within the yellow area which denotes the alpine domains of the Adige, Brenta, Piave, and Tagliamento catchments defined by outlet points (red circles) located in the southern foreland basin. Major rivers are annotated and major cities are indicated by white squares. The position of the Periadriatic fault system, Valsugana thrust, and Fella fault is shown (solid red line), and individual fault segments are annotated. Purple arrows indicate the position of elbow‐shaped bends of major rivers. Green and yellow stars indicate the position of T‐shaped river junctions and prominent wind gaps, respectively. The current position of the Euler Pole of the Adriatic plate is shown (black star with yellow outline), and black arrows indicate variations in the current convergence rate.

All processes mentioned above have heavily influenced the topographic evolution of the tectonically active eastern part of the European Alps. The topographic evolution of the Eastern Alps (EA) and eastern Southern Alps (ESA) is controlled by the Late Oligocene–Early Miocene indentation [*Handy et al*., 2015] of the Adriatic microplate into an overthickened orogenic wedge emplaced on top of the European plate. The Adriatic indenter (i.e., the ESA) and the EA are separated by the Periadriatic fault system (Figure 1). Indentation caused north‐south shortening, crustal thickening, and consequently surface uplift and promoted horizontal advection of crustal fragments along orogen‐scale strike‐slip faults [e.g., *Ratschbacher et al*., 1991a, 1991b; *Frisch et al*., 1998; *Linzer et al*., 2002]. Crustal deformation seems to be unequally partitioned between the presumably stiffer, less deformed Adriatic indenter (ESA) and the presumably softer, more deformed orogenic wedge (EA) [*Ratschbacher et al*., 1991b; *Willingshofer and Cloetingh*, 2003; 2008a, 2008b]. However, there is evidence that the accommodation of crustal deformation distributed between the two domains changed over time and that the formation of alpine topography in the ESA initiated mainly in Middle‐Late Miocene times with the activation of the Valsugana thrust and the Fella strike‐slip fault [*Castellarin and Cantelli*, 2000; *Zattin et al*., 2006]. Whatever may have caused the shift of deformation from the EA to the ESA, the ongoing shortening of the Adriatic indenter accompanied by the reconfiguration of topography in this domain is expressed by numerous seismic events [*Galadini et al*., 2005; *Burrato et al*., 2008]. In contrast, the domain of the EA is characterized by low seismicity. Differences in timing and rates of crustal deformation in the two domains imply that topography buildup and landscape dissection differ significantly between the ESA and the EA. Nevertheless, landscape evolution of both domains is linked via the ESA‐EA drainage divide (Figure 1).

Drainage systems of the ESA and EA are known to have reacted sensitively to tectonic and climatic signals of the last few million years as indicated by the migration of drainage divides, river capture events, and knickpoints [*Dunkl et al*., 2005; *Kuhlemann et al*., 2006; *Robl et al*., 2008a; *Monegato and Stefani*, 2010; *Monegato et al*., 2010; *Monegato and Vezzoli*, 2011]. The uneven partitioning of deformation between the EA and the ESA including the latest Middle Miocene to Late Miocene shift of deformation to the eastern Adriatic indenter (with subsequent propagation toward the southern foreland basin) [e.g., *Castellarin and Cantelli*, 2000; *Zattin et al*., 2006] and Late Miocene base level changes in the Mediterranean [e.g., *Roveri et al*., 2003; *Willett et al*., 2006; *Mancin et al*., 2009] and the northern Molasse Basin [e.g., *Gusterhuber et al*., 2012; *Baran et al*., 2014] implies that drainages of these two distinct alpine domains should differ in their channel metrics, network topology, and state of maturity. Consequently, evidence of active landscape adjustment should be reflected in geometric properties of rivers and torrents of the Adriatic indenter, and of watersheds separating the two domains. However, fluvial topographic patterns may be altered by the impact of the Pleistocene glaciations complicating the interpretation of channel metrics in terms of tectonic forcing and far‐field effects.

The topography of the ESA has not been studied with respect to the interplay of crustal deformation, base level changes, and erosional surface processes. This is in contrast to the Eastern Alps, north of the Periadriatic fault, where geomorphological studies [e.g., *Robl et al*., 2008a; *Hergarten et al*., 2010; *Wagner et al*., 2011; *Legrain et al*., 2014a, 2014b; *Robl et al*., 2015; *Dixon et al*., 2016] led to a detailed understanding of alpine landscape evolution since the pioneering studies of *Frisch et al*. [1998] and *Szekely et al*. [2002].

In this study, we contribute to closing this knowledge gap and systematically analyze characteristic metrics of four major catchments draining the eastern Adriatic indenter east of the Giudicarie fault (Figure 1) and discuss these metrics in terms of (a) crustal deformation driving uplift and horizontal advection, (b) lithology‐controlled bedrock resistance to fluvial incision, and (c) base level changes as a consequence of the Messinian salinity crisis and the inversion of the Molasse Basin, and glacial carving during the Pleistocene.

## 2 Study Region

The study region encompasses four major rivers draining the eastern Adriatic indenter (in the sense of *Handy et al*. [2015]), i.e., the still northward moving triangular northeastern part of the Southalpine block that indented the Eastern Alps since circa 21 Ma. These are from west to east the Adige, Brenta, Piave and Tagliamento Rivers. Their drainage networks are roughly confined by the sinistral transpressive Guidicarie fault, the dextral transpressive Periadriatic fault, and the Venetian‐Friulian Plain in the west, north, and south, respectively (Figure 1). The eastern termination of the study region coincides with the drainage divide between the Tagliamento River in the west and the Sava River‐Soca/Isonzo River in the east. The Soca/Isonzo catchment is not considered due to a lack of high‐resolution digital elevation models. The evolution of individual drainage systems leads to growth and shrinkage of catchments by drainage divide migration and river piracy events and controls the orogen‐scale topographic pattern [e.g., *Cotton*, 1958; *Stüwe et al*., 2008; *Bonnet*, 2009; *Willett et al*., 2014; *Yang et al*., 2015]. The Eastern and Southern Alps feature two major west‐east trending drainage divides. The main divide follows roughly the highest peaks of the Alps, separating the drainages of the Inn River, Salzach River, and Enns River in the north from the Drau River und Mur River in the south (Figure 1). Major Alpine rivers from both sides of the watershed are tributaries of the Danube River, and the Black Sea represents the common base level. A second major drainage divide several tens of kilometers south of the main divide follows the spur of the eastern Adriatic indenter. All major rivers of the ESA drain toward the south following the general topographic gradient of the indenter and share the Mediterranean Sea as their base level. The varying degree of landscape dissection in the ESA and EA is mostly attributed to these independent base levels with a major incision event following the Messinian desiccation of the Mediterranean Sea [*Roveri et al*., 2003; *Manzi et al*., 2005; *Loget et al*., 2006; *Willett et al*., 2006; *Robl et al*., 2008a; *Monegato et al*., 2010; *Preusser et al*., 2010] but may also be the expression of the different tectonic histories of the two alpine realms. However, both effects should be recorded by the incision of rivers into their bedrock and by the position of major watersheds.

### 2.1 Major Catchments Draining the ESA

**Figure 2:**
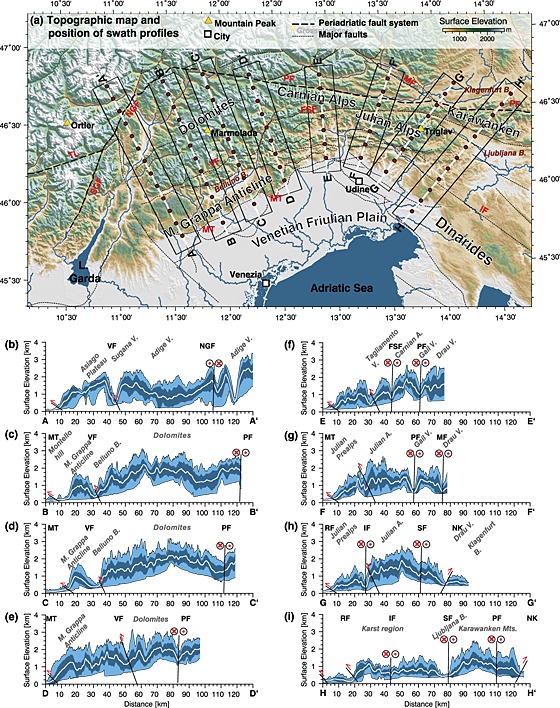
Eight swath profiles across the ESA orientated normal to the orogenic front. The swath profiles start within the southern foreland basin and end west and north of the Periadriatic fault system (PF), respectively. (a) The position of the swath profiles is indicated by black rectangles placed on a topographic map. The Periadriatic fault system and other major faults are represented by dashed and dotted black lines, respectively. Major mountain peaks (yellow triangles with black outline), cities (white squares with black outline), major rivers (blue solid lines) and lakes, and the Adriatic Sea (blue areas) are shown for reference. Swath profiles are 20 km wide and up to 120 km long. The distance is split into 10 km segments (red circles) to allow a direct linkage of topographic features on the map with topographic swath profiles AA′ to HH′. The curves in swath profiles comprise the mean elevations (white line), extreme values (envelope of the light blue area), and mean values ± 1 standard deviation (envelope of the dark area). Faults and their shear sense are indicated (circles with red crosses and dots), and important topographic features are annotated consistently in the map and the profiles. Abbreviations: FSF, Fella‐Sava fault; IF, Idrija fault; MT, Montello thrust; MF, Möll valley fault; NGF, North Giudicarie fault; NK, North Karawanken Thrust; PF, Periadriatic fault; SGF, South Giudicarie fault; TL, Tonale fault; and VF, Valsugana fault.

The Adige, Brenta, Piave, and Tagliamento Rivers drain the largest part of the ESA. In the eastern sector of the study region, the watershed separating the Mediterranean Sea and the Black Sea base levels roughly follows the Pustertal‐Gailtal segment of the Periadriatic fault system but is offset by several kilometers to the south (Figures 1 and 2). Only in the western sector, at the northwestern rim of the eastern Adriatic indenter, a number of tributaries of the Adige River spread to the realm of the EA north of the Pustertal fault and west of the Giudicarie fault (Figure 1). The Adige River itself and its western tributaries feature peculiar elbow‐shaped channel segments at intersections with the Giudicarie fault (Figure 1, magenta arrows). These features are consistent with sinistral motion along this fault system suggesting that rivers of the ESA are advected by faults.

Orogen‐parallel flow is prominent within the eastward extruding wedge north of the indenter (e.g., Enns River and Salzach River, Figure 1). In the ESA, channel segments featuring orogen‐parallel flow occur to a lesser extent, mostly in the uppermost reaches of the drainage systems. The easternmost tributary of the Adige River, the Rienz River, flows strike parallel to the Pustertal fault segment of the Periadriatic fault system from east to west and therefore against the general topographic gradient of the orogen (Figure 1). This indicates that faults can impose flow directions on major rivers. Large orogen‐parallel channel segments with T‐shaped sections are observed in the Tagliamento and also in the Soča/Isonzo drainage system. In both cases, two tributaries drain the same lineament in opposite direction and turn by approximately 90° at their confluence point (Figure 1, yellow stars). In combination with prominent wind gaps (Figure 1, green stars), this is a characteristic feature of river piracy—a process proposed for several rivers of the Eastern Alps (e.g., Mur River‐Mürz River [*Robl et al*., 2008a; *Wagner et al*., 2011]) and Southern Alps (e.g., Tagliamento River‐Fella River [*Monegato and Vezzoli*, 2011]). A similar pattern is observed at the orogen side of growing anticlines in the peripheral foreland basins of the Alps [*Benedetti et al*., 2000] and the Himalayas [*Delcaillau et al*., 2006; *Sakai et al*., 2006] and is caused by damming and redirecting of major rivers. *Monegato et al*. [2010] documented the succession of river piracy events due to the north directed migration of the Tagliamento watershed in the last few million years and reported bypassing of the Piave River at the Monte Grappa anticline (Figure 2). North of the Monte Grappa anticline, the Piave catchment features a highly asymmetric drainage system as the Piave River drains along the Valsugana fault near its eastern drainage divide, and all major tributaries originate west of the receiving stream.

Clearly, the drainage network of the ESA shows the deformation history of the region. However, prominent morphological features such as glacial lakes (e.g., Lake Garda) indicate that the Pleistocene glaciations have reshaped the preglacial alpine landscape by the formation of glacial landforms and even reorganized the course of ESA rivers [e.g., *Garzanti et al*., 2011; *Monegato and Vezzoli*, 2011; *Fontana et al*., 2014].

### 2.2 Topographic Features and Structural Elements

In general, alpine topography and the position and orientation of valleys drained by major rivers are controlled by brittle structures that have accommodated shortening of the ESA since Middle Miocene–Late Miocene times [*Castellarin et al*., 2006; *Caputo et al*., 2010]. The topography of the ESA largely mimics the structural framework of southward propagation of thrusting during the evolution of the Southalpine fold‐and‐thrust belt [*Castellarin and Cantelli*, 2000]. Exhumation of deep crustal material was confined to localized areas of rapid growth, migration, and erosion of the fold‐and‐thrust belt, e.g., along the Valsugana thrust [e.g., *Zattin et al*., 2006]. The topographic segmentation of the ESA is documented by eight swath profiles with starting and ending points in the Venetian‐Friulian Plain and the EA, respectively (Figure 2). All swath profiles are aligned roughly normal to the orogenic front. They show similar topographic features related to thrusts, back thrusts, and strike‐slip faults but show distinct peculiarities in the western (profiles A–D) and eastern sectors (profiles E–H). In the western sector topography rises rapidly at the hanging wall of the thrusts near the transition from the Venetian‐Friulian Plain to the orogen with peak elevations exceeding 2000 m (Figure 2, swath profiles A–D). This southernmost alpine area is confined in the north by several fault‐related basins such as the Sugana Valley or Belluno Basin. The highest mean elevations along the swath profiles occur in the central parts of the Dolomites approximately at middistance between the Valsugana (VF) and Fella‐Sava (FSF) fault systems. In this region, peak elevations exceed 3000 m. Thus, the Dolomites act as a local watershed separating northward and southward draining streams.

The eastern sector of the ESA is also characterized by a distinct transition from the foreland basin to mountainous topography (Figure 2, swath profiles E–H). Topography rises at the hanging walls of several thrusts (e.g., Julian Prealps) but is disconnected by a series of parallel, west‐east to northwest‐southeast directed fault‐related valleys. This range‐valley‐range setting causes high local relief. Highest mean elevation and peak elevations are observed in the sliver‐shaped Carnian Alps that are wedged between the Fella‐Sava fault and the Periadriatic fault system, and farther east in the clast‐shaped Julian Alps that are confined by the Idrija fault and the Fella‐Sava fault in the south and north, respectively.

### 2.3 Climatic and Tectonic Constraints on the Evolution of Indenter Topography

**Figure 3:**
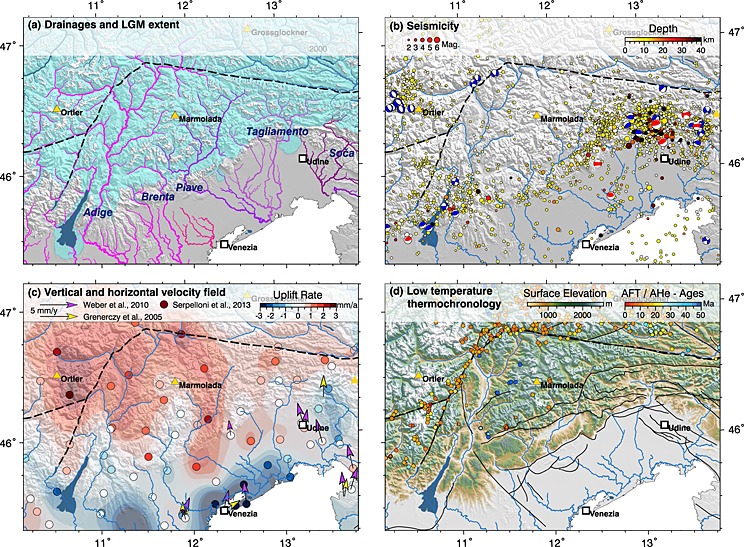
Climatic and tectonic constraints on the evolution of alpine topography. Important peaks (yellow triangles), major cities (white squares with a black outline), the position of the Periadriatic fault system (black dashed lines) and major rivers (blue lines) are shown for reference. (a) Extent of glaciers during the last glacial maximum [*Ehlers et al*., 2011] (blue area) and important drainages systems of the Eastern Alps color coded for their base levels as displayed in Figure 1. Major rivers of the Southern Alps are annotated. (b) Spatial position of recent seismic events compiled by IRIS (http://www.iris.edu/) indicated by circles that scale in size with the magnitude and are color coded for the depth of the hypocenters. The tectonic regime is indicated by centroid moment tensors (ISC‐GEM catalogue) [*Storchak et al*., 2013] (beach balls with blue compressional sectors) and from the centroid moment tensor project [*Dziewonski et al*., 1981; *Ekström et al*., 2012] (beach balls with red compressional sectors). (c) Vertical (filled circles) and horizontal (arrows) velocity field based on GPS time series indicating recent crustal motion and deformation in the ESA [*Grenerczy et al*., 2005; *Weber et al*., 2010; *Serpelloni et al*., 2013]. (d) Compilation of low‐temperature thermochronology data based on the distribution of apatite fission track ages (circles) [e.g., *Luth and Willingshofer*, 2008 and references therein; *Fox et al*., 2016, and references therein] and apatite (U–Th–Sm)/He ages (squares) [*Heberer et al*., 2016]. Solid black lines indicate the position of major faults.

Similar to the Eastern Alps, the landscape of the Adriatic indenter was affected by glacial erosion during the Pleistocene glaciations (Figure 3a). Only the southern foothills and the southern foreland basin were not occupied by glaciers and remained unaffected by direct glacial erosion or deposition [*Fontana et al*., 2014]. Terminal moraines document at least three piedmont glaciers that advanced toward the foreland basin during the last glacial maximum (LGM) [*Ehlers et al*., 2011; *Fontana et al*., 2014]. The largest piedmont glacier of the Adriatic indenter was located at Lake Garda. There is evidence for redirection of major streams of the ESA related to the Pleistocene glaciations with few documented stream capture events but the course of most rivers remained unaffected [*Monegato et al*., 2010; *Garzanti et al*., 2011]. However, hanging valleys and knickpoints may be attributed to glacial erosion responsible for the perturbation of drainage networks in the Alps [e.g., *Penck*, 1905; *Robl et al*., 2008a; *Norton et al*., 2010; *Valla et al*., 2010].

The ESA are one of the tectonic hot spots of the European Alps. Crustal shortening is expressed by frequent seismic events [*Galadini et al*., 2005; *Cheloni et al*., 2012, 2014] and active formation of topography (Figure 3b). In particular, the southern foothills at the transition from the orogen to the foreland basin are affected by numerous earthquakes. Fault plane solutions reveal thrusting as dominant mechanism of deformation [*Dziewonski et al*., 1981; *Willingshofer and Cloetingh*, 2003; *Galadini et al*., 2005; *Ekström et al*., 2012; *Storchak et al*., 2013]. Striking morphological features at the mountain front like growing anticlines or abandoned valleys are spatially and structurally linked to back thrusts and strike‐slip zones [*Benedetti et al*., 2000]. Active deformation west of Lake Garda, at the western indenter tip and at the South Alpine‐Dinaric junction, is indicated by numerous earthquakes (Figure 3b). There, the north‐south extent of the deformed Adriatic indenter is minimal. In contrast, the most elevated region of the indenter appears seismically calm—an observation also made for the Tauern Window region north of the Adriatic indenter hosting some of the highest peaks of the Eastern Alps (Figures 1 and 3b).

Gradients in the horizontal and vertical velocity field are responsible for the advection of rivers and alter the geometric properties of drainage basins, which in turn trigger landscape adjustment [e.g., *Hallet and Molnar*, 2001; *Yang et al*., 2015]. The horizontal velocity field of the Southern Alps and adjacent regions indicates the counterclockwise rotation of Adria around an Euler pole now located near Milano with an angular velocity of 0.52 mm yr^−1^ [*Nocquet and Calais*, 2003] (Figures 1 and 3c). This results in a north directed velocity of 3 mm yr^−1^ at the southern undeformed Adriatic indenter near the cities of Venice and Trieste and a decrease of horizontal motion within the orogen where plate convergence is progressively compensated by crustal shortening [*Grenerczy et al*., 2005; *Weber et al*., 2010] (Figures 3b and 3c). North of the indenter, east directed motion (lateral extrusion) on the order of 1 mm yr^−1^ is measured [*Grenerczy et al*., 2005]. The vertical velocity field in the ESA reflects crustal shortening but may be influenced by erosion‐ [e.g., *Champagnac et al*., 2007] and deglaciation‐induced isostatic rebound. The latter mechanism fades out rapidly with the waning of alpine glaciers [*Norton and Hampel*, 2010], while the former may still contribute to uplift. Catchment wide erosion rates from the ESA do not exceed 0.4 mm yr^−1^ [*Norton et al*., 2011], which is about 1 order of magnitude lower than GPS‐derived uplift rates. Although GPS time series are short, the derived horizontal velocity field is consistent with the orientation of active faults and the vertical velocity field is roughly correlated with the topography of the Alps [*Serpelloni et al*., 2013] (Figure 3c). The ESA and the adjacent foreland basin feature strong gradients in the vertical velocity field with subsidence above 3 mm yr^−1^ near Venice (Venetian platform) and uplift beyond 2 mm yr^−1^ in the headwaters of the Adige River, Piave River, and Tagliamento River [*Cenni et al*., 2013; *Serpelloni et al*., 2013; *Cheloni et al*., 2014]. Low subsidence rates are reported for the lower Adige River, while the Brenta River and the Piave River pass a zone of high‐uplift rates. The Tagliamento valley is characterized by moderate uplift rates, except for one GPS station near Tolmezzo that reports low subsidence rates.

Low‐temperature thermochronology yields rates and duration of cooling and exhumation and can thus be used to constrain timing and rates of erosional surface processes [e.g., *Reiners and Brandon*, 2006]. However, the scarcity of suitable lithology limits the applicability of low‐temperature thermochronometers for the Adriatic indenter, and data are mainly focused along major thrust systems (e.g., Valsugana thrust). There, latest Middle Miocene to Late Miocene exhumation and unroofing of ~ 4 km has led to the exposure of crystalline basement [*Zattin et al*., 2006; *Heberer et al*., 2016]. In contrast, apatite fission track ages to the north and immediately to the west of the Valsugana thrust are Mesozoic [*Zattin et al*., 2003; *Emmerich et al*., 2005; *Heberer et al*., 2016], corroborating that overall exhumation throughout most of the ESA is minor [*Emmerich et al*., 2005; *Zattin et al*., 2006] (Figure 3d). Thin‐skinned tectonic shortening with fault‐propagation folding and fault‐bend folding prograded southward in the ESA, producing a pronounced relief, e.g., of 1200 m above the plain along the SE verging Bassano‐Valdobbiadene thrust [*Galadini et al*., 2005] (Figure 5). The main phase of shortening and exhumation in the ESA succeeded a phase of strain localization and major exhumation restricted to the EA in the Tauern Window. Such a shift from focused exhumation in front of the indenter (Tauern Window) to more distributed exhumation within, as well as deformation of the indenter, may be related to a major shift in the coupling state between the European plate and the Adriatic indenter during Late Miocene times, from a decoupled to a coupled system [*Willingshofer and Sokoutis*, 2009; *Heberer et al*., 2016]. The stratigraphic record of the EA reveals the highest overall exhumation at the northern and western indenter margin with the outcrops of metamorphic rocks belonging to the Southalpine basement, Paleozoic sediments, and the Permian volcanics (Figure 5a). Toward the south the sedimentary cover is getting increasingly younger and features carbonatic and clastic rocks from Triassic to Lower Cretaceous age. Sediments of Miocene age occur at recently uplifted anticlines that represent the southernmost topographic highs of the Adriatic indenter within the southern foreland basin [*Benedetti et al*., 2000]. In the upper reaches of the drainage system, continuous surface uplift since the Messinian is recorded by uplifted terraces of the Tagliamento River [*Monegato and Stefani*, 2010].

Despite this wealth of information on the geodynamic evolution and the present tectonic regime of the EA‐ESA realm, a number of questions regarding the interaction of crustal deformation, erosion, and climate in shaping the present‐day topography remain: (1) Which processes shaped the present‐day topography of the Adriatic indenter and adjacent domains? (2) How are ESA channel metrics related to uplift rates, lithology, and glaciation? (3) How did base level changes in the Mediterranean and the northern foreland basin affect the ESA channels and the EA‐ESA drainage divides?

## 3 Data and Methods

Our morphometric approach uses the TINITALY digital elevation model (DEM) that covers the entire Italian territory [*Tarquini et al*., 2007, 2012] and a lidar‐based DEM released by the Federal State Government of Austria as part of the INSPIRE (Infrastructure for Spatial Information in the European Community) initiative. These data sets have a spatial resolution of 10 m. Shuttle Radar Topography Mission (SRTM) data with a spatial resolution of 3 arc sec were used for orogen‐wide morphometric analyses.

### 3.1 Drainage Pattern Extraction

Data preprocessing included the per cell computation of flow direction (D8) and contributing drainage area, as well as the determination of drainage divides, and was based on standard methods of GRASS GIS [*Neteler et al*., 2012]. The drainage area cutoff for the definition of the flow path and the drainage network was set to 1 km^2^. Based on DEM cells exceeding this threshold, longitudinal channel profiles and the topology of the drainage network were calculated individually for all large catchments of the study area. To include tributaries draining the southern fringe of the ESA, coordinates of outlet points for all large north‐south draining channels were defined some tens of kilometers south of the orogenic front. We did not consider streams draining the southern foreland basin as low topographic gradients, and strong anthropogenic impact leads to the calculation of ambiguous flow directions.

To obtain channel profiles we follow the main channel (Hack's Order 0), defined by the largest contributing drainage area, in the upstream direction and store the three‐dimensional geometry of the channel. Starting from the main channel, we mark each river confluence as outlet point of the channel of higher Hack Order and iteratively repeat this approach until the topology of the entire drainage system is resolved. In consequence of our approach for extracting channel profiles, the flow path with Hack's channel order 0 does not necessarily coincide with the eponymous river of the catchment (e.g., Adige catchment: the contributing drainage area of the Eisack River is larger than the contributing drainage area of the Adige River). For better comparison, channel profiles are reversed and presented as downstream profiles with channel distance *x* = 0 km at the origin of the rivers. To reduce the noise introduced by the digital elevation model and to obtain exclusively monotonous profiles, we resample the raw channel profiles to a minimum vertical distance of 5 m between individual vertices. The computation of characteristic channel metrics (e.g., channel slope) is based on these smoothed channel profiles.

### 3.2 Model Description

In order to explore deviations of the drainage system from an equilibrium state defined by constant rock uplift rates and erosional response in a uniform lithology, we apply the most frequently used detachment‐limited model for bedrock channel incision [*Howard*, 1980, 1994; *Hergarten*, 2002] of the form

(1) $$\frac{\partial H}{\partial t}=U-KA^{m}{\left(\frac{\partial H}{\partial x}\right)}^{n}$$

where *H* is elevation, *t* is time, and *x* is the longitudinal coordinate along the river profile increasing in the upstream direction. *U* and *K* stand for uplift rate and rock erodibility, respectively. *A* represents the contributing drainage area as proxy for the discharge of a river, and $\frac{\partial H}{\partial x}$ is the channel slope where *m* and *n* are exponents describing the nonlinear contribution of channel slope and drainage area to river incision. This mathematical description for fluvial incision roots on the empirical relationship discovered by *Hack* [1957] that the channel slope is inversely proportional to the upstream drainage area to the power *θ*, where *θ* is the concavity index, equal to m/n [*Flint*, 1974].

In our morphometric approach, we compare natural drainage systems with a topographic steady state where $\frac{\partial H}{\partial t}=0$ and equation (1) simplifies to

(2) $$\frac{\partial H}{\partial x}={\left(\frac{U}{K}\right)}^{\frac{1}{n}}A^{-\frac{m}{n}}$$

If uplift rate and bedrock erodibility are constant, we get

(3) $$\frac{\partial H}{\partial x}\propto A^{-\theta }$$

Under these assumptions the product

(4) $$k_{s}=\frac{\partial H}{\partial x}A^{\theta }$$

is constant, where *k_s_* is the steepness index [e.g., *Snyder et al*., 2000; *Wobus et al*., 2006] and directly represents the ratio between *U* and *K* for *n* = 1. As even the physical dimension of *k_s_* depends on the value of *θ*, steepness indices of different rivers or river segments can only be compared if the same reference value *θ*_ref_ is used for all channels of the investigated area. The respective values of *k*_s_ are denoted normalized steepness index *k*_sn_ and can be interpreted in terms of variable uplift rates, contrasting lithology, or time‐dependent reorganization of the drainage system toward a new equilibrium [*Whipple and Tucker*, 1999; e.g., *Whipple et al*., 2013, and references therein]. Whenever we confront measured with modeled equilibrium channel profiles, the concavity index becomes a crucial factor as it controls the profile curvature. The range of *θ* was determined by *Hack* [1957] with lower and upper limits of 0.25 and 0.6, respectively, but empirical observations and theoretical arguments show that *θ* = 0.5 or slightly below (*θ* = 0.45) is expected for bedrock channels in fluvial equilibrium [e.g., *Whipple and Tucker*, 1999; *Wobus et al*., 2006]. To avoid odd units for *k*_sn_, we employ *θ*_ref_ = 0.5.

Changes in uplift rate or contributing drainage area recorded in the channel geometry are obscured by the curvature of the channel profile that is introduced by the nonlinear decrease of contributing drainage area (being a proxy of discharge) with the upstream distance. To overcome this problem, we apply the recently introduced *χ* transformation [*Perron and Royden*, 2013; *Royden and Perron*, 2013], where the longitudinal coordinate *x* is transformed to a new coordinate *χ* so that the curvature of the equilibrium channel profile vanishes. Contributing drainage area can be eliminated from Flint's law if the transformation satisfies the condition

(5) $$\frac{dx}{d\chi }={\left(\frac{A}{A_{0}}\right)}^{\theta }$$

which is achieved by

(6) $$\chi =\int_{x_{0}}^{x}{\left(\frac{A}{A_{0}}\right)}^{-\theta }dx$$

where *x*_0_ is an arbitrary reference point, and *A*_0_ is an arbitrary reference catchment size (*A*_0_ = 1 km^2^) introduced to avoid odd physical dimensions for *χ*.

Equilibrium channel profiles under spatially uniform climatic and tectonic conditions are straight lines in the coordinate system *χ* versus *H* with the slope of the line proportional *k_s_*. Deviations can therefore be interpreted in terms of temporal or spatial variations of climatic conditions or tectonic forcing [e.g., *Whipple et al*., 2013], or changes in drainage area due to the long‐term reorganization of the drainage network [*Goren et al*., 2014]. We calculated the *χ* values for all channels with a contributing drainage area > 1 km^2^ by employing a novel numerical tool [*Hergarten et al*., 2016]. We compare channel profiles of major streams in the *χ* space and present channel segments color coded according to their *χ* values. Such *χ* maps are useful indicators to constrain the stability of drainage divides and the direction of drainage divide migration [*Willett et al*., 2014]. Differences in the *χ* values at both sides of a drainage divide indicate either a spatial variation in uplift rate (or erodibility) or a disequilibrium where the drainage divide migrates. *Willett et al*. [2014] presented an extension of the *χ* method toward inhomogeneous uplift rates, but this extended transform requires that the uplift rate is known within the entire region or at least along a river profile. If the uplift is not known, differences in *χ* across a drainage divide can be directly interpreted in terms of different mean erosion rates using the following approach. According to equations (1) and (5), the erosion rate is

(7a) $$E=KA^{m}{\left(\frac{\partial H}{\partial x}\right)}^{n}$$

(7b) $$=KA_{0}^{m}{\left(\frac{\partial H}{\partial \chi }\right)}^{n}$$

so that

(8) $$\frac{\partial \chi }{\partial H}=A_{0}^{\theta }{\left(\frac{K}{E}\right)}^{\frac{1}{n}}$$

Integrating this equation from a given base level leads to

(9) $$\chi =A_{0}^{\theta }\int {\left(\frac{K}{E}\right)}^{\frac{1}{n}}dH+\text{const}$$

This relationship allows for a direct interpretation of differences in *χ* at both sides of a drainage divide in terms of the mean ratio *K*/*E*. Averaging is performed along the vertical axis (integration over *H* instead of *x* or *χ*). For a nonlinear stream power law (*n* ≠ 1) the average of the function ${\left(\frac{K}{E}\right)}^{\frac{1}{n}}$ is taken. The side of the drainage divide where *χ* is larger must be characterized either by higher erodibilities or by lower actual erosion rates in the mean, regardless of whether the latter arise from lower uplift rates or from disequilibrium.

## 4 Possible Scenarios of Orogen Evolution and Divide Migration in the Alps

In order to disentangle various processes that drive ESA‐EA landscape evolution by geomorphic properties, we demonstrate the impact of lithology, uplift rate, base level change, and glacial erosion on a simplified one‐dimensional orogen. The simplified orogen consists of two channels separated by a drainage divide that can migrate toward the steeper channel as a consequence of hillslope diffusion. To describe the time‐dependent evolution of channels characterized by a detachment‐limited regime, we numerically solve equation (1) with *n* = 1 and an additional diffusion term for various initial and boundary conditions. As a one‐dimensional model does not allow for the computation of catchment sizes, we used the power law relationship between catchment size and upstream length toward the drainage divide *l* originally found by *Hack* [1957], *l* ∝ *A* ^*h*^, with *h* = 0.56. Then the term *A^θ^* in equation (1) turns into $l^{\frac{\theta }{h}}$ except for a constant factor, which can be included in the erodibility *K*.

**Figure 4:**
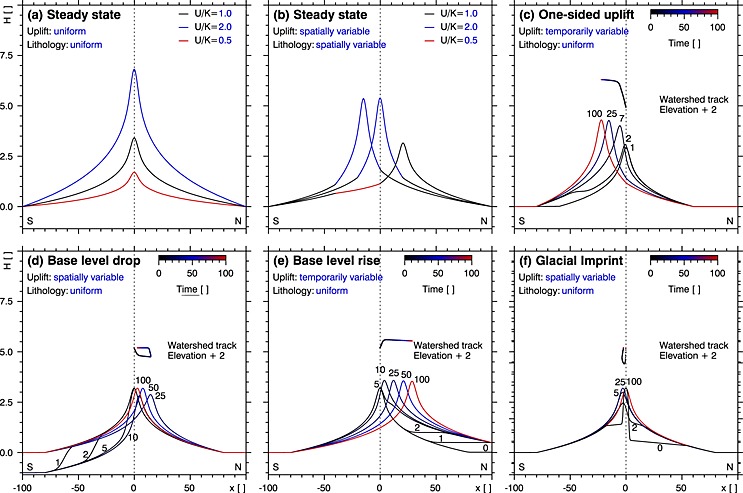
State and evolution of channels and drainage divide of a simplified orogen calculated for several scenarios: Steady state (a) assuming uniform uplift rate (*U*) and uniform bedrock erodibility (*K*) and (b) spatially variable *U* and/or *K* indicated by different line colors; time‐dependent evolution of alpine topography assuming (c) a pulse of additional uplift south of the main divide to imitate Middle to Late Miocene uplift in the ESA; (d) a massive, sudden, and temporally limited base level drop between *t* = 0.1 and *t* = 20 in the south to mimic the Messinian salinity crisis; (e) a continuous base level rise in the north representing the inversion of the northern foreland basin; and (f) the transition from a glacially shaped landscape with low‐gradient valleys and oversteepened valley heads to a fluvial landscape. Note that we mimic the impact of glacial erosion on preglacial steady state valleys by limiting the valley slope within an arbitrarily defined vertical range. Channel profile time series are color coded and annotated for dimensionless time. The watershed track (color coded for time) traces the vertical and horizontal position of the watershed over time and is shifted by 2 length units in vertical direction for a clearer representation. All time‐dependent scenarios are characterized by zero uplift zones at the model boundaries (flat channel segments) to mimic forelands or marine domains. Note that the model results are dimensionless but may be dimensionalized by applying appropriate scaling parameters for length, uplift rate, and rock erodibility.

For steady state with uniform uplift rate (*U*) and bedrock erodibility (*K*), neglecting precipitation contrasts, the channels are symmetric on both sides of a watershed at middistance between the stable base levels at the southern and northern boundary of the model domain (Figure 4a). Channel gradients as well as peak elevation are a linear function of *U*/*K*, where high and low *U*/*K* ratios lead to high and low peak elevations, respectively. In case of spatial variations of *U*/*K*, channels feature anchored knickpoints with channels steepening with increasing *U*/*K* (Figure 4b). The range may become asymmetric with respect to the stable base levels as the divide migrates toward high *U*/*K* and away from low *U*/*K* zones. In such a case, the drainage divide is stable, although the flow length, and hence the average channel gradient of the channel north and south of the drainage divide, differs.

Deformation of a continental indenter due to a changing tectonic regime may cause increased uplift rates at the peripheral parts of the orogen (Figure 4c). In this scenario, the uplift rate at the southern half of a formerly steady state orogen is increased by 50% of the initial uplift rate. This results in channel steepening via migrating knickpoints. The drainage divide shifts toward higher uplift rates and the peak elevation increases. The model shows that the increase of peak elevation is rather fast compared to the horizontal shift of the watershed to a stable position. A sudden one‐sided base level drop such as the Messinian salinity crisis results in a very steep channel section migrating upstream (Figure 4d). The lowering of the southern channel causes a shift of the drainage divide toward the north and a reduction of the peak elevation. The divide is stable until the signal of the base level drop reaches the main divide, which causes some delay between the base level drop and the initiation of the divide migration. The position of the drainage divide and the peak elevation are restored after the base level returns to the initial state. If the base level drop is short lived, most of the lowered channel section becomes buried and only a fraction of the oversteepened channel migrates upstream and causes only a minor and temporally limited shift of the divide. A permanent one‐sided rise of the base level such as the inversion of the northern foreland basin leads to a progressive shift of the watershed toward the north without significant changes in peak elevation (Figure 4e). The Pleistocene glaciations altered the topography of midlatitude mountain ranges such as the European Alps. (Figure 4f). Glacial erosion leads to low‐gradient valley sections and steep valley heads characteristic of alpine landscapes with a significant glacial imprint. The postglacial adjustment of such a glacially perturbed orogen causes the vanishing of low‐gradient channel sections due to migrating knickpoints. Glacially oversteepened ridges and horns decay, leading to a temporally limited reduction of peak elevation (not considering isostatic rebound). For the case of stronger glacial erosion north of the divide, the watershed is temporally shifted to the south (Figure 4f).

After formulating possible scenarios of orogen evolution and divide migration and presenting related topographic signatures, we inspect the morphometry of the ESA and the ESA‐EA drainage divide to decipher processes constructing and destructing topography and constrain possible climatic and tectonic drivers.

## 5 Results

### 5.1 Variations in *k*_sn_

**Figure 5:**
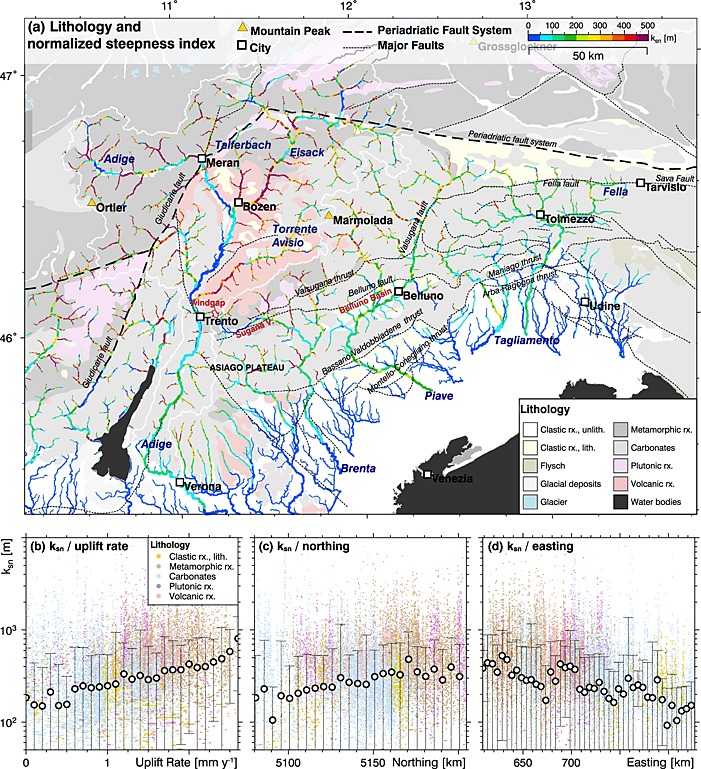
(a) The normalized steepness index (*k*_sn_) of rivers superposed on a simplified geological map of the study region based on the Geological Map of Italy 1:1,250,000; frontal thrusts were taken from *Burrato et al*. [2008]. Southalpine drainage system is color coded for *k*_sn_ applying a concavity index *θ* = 0.5. All channels with a contributing drainage area *A* larger 1 km^2^ are shown. The line width of the channels is proportional to log_10_ (*A*). Drainage divides are indicated by black outlines, and major catchments are annotated. (b–d) *k*_sn_ plotted against uplift rate [*Serpelloni et al*., 2013] (bin size = 0.1 mm yr^−1^), northing (bin size = 5 km), and easting (bin size = 5 km), respectively. All channels with a contributing drainage area *A* larger 10 km^2^ are considered here. White dots and error bars indicate bin average and standard deviation; raw point clouds are color coded for bedrock lithology. Note that large standard deviations in *k*_sn_ result from the inherent roughness of channel profiles that plague all morphometric methods relying on channel slopes (e.g., the determination of *θ* from the relation between catchment size and channel slope). The coefficient of correlation between *k*_sn_ and uplift rate is 0.17.

**Figure 6:**
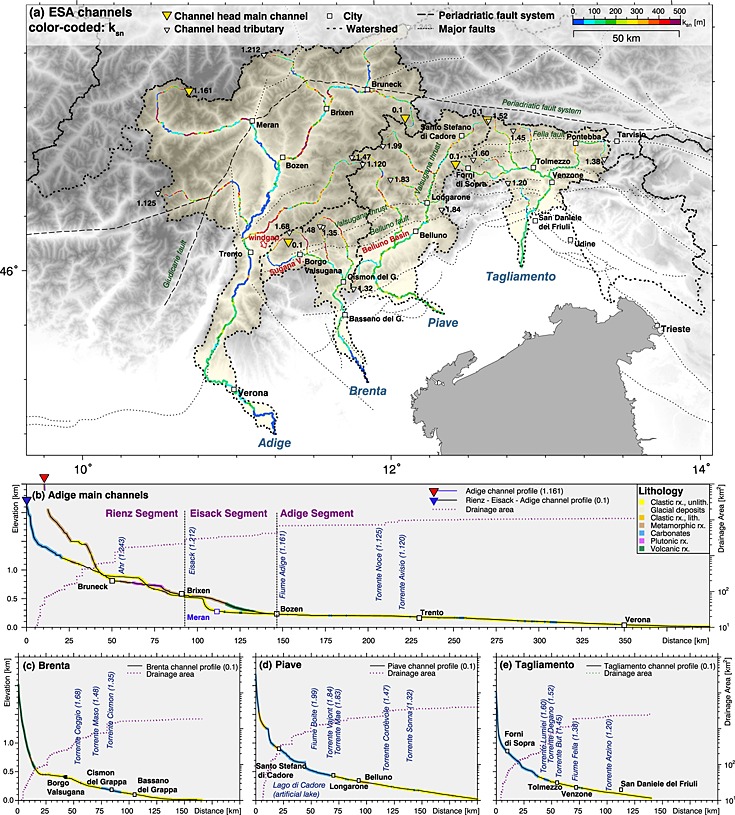
Channel metrics of ESA rivers. (a) Topographic map of four major ESA catchments presenting *k*_sn_ values for the main channels and important tributaries. Longitudinal channel profiles of the (b) Adige River, (c) Brenta River, (d) Piave River, and (e) Tagliamento River. The lithology of the channel bed is color coded. Channel heads of main channels shown in Figures 6b–6e and major tributaries are indicated by yellow and white triangles, respectively. Channel order and ID are annotated and separated by comma (e.g., 1.120 means the 120th channel of Hack's stream order 1 numbered in the upstream direction).

For a given uplift and lithology and assuming that erosion can be described by the stream power law, the normalized steepness index (*k*_sn_) is a measure of the ability of a river to incise into its bedrock. Consequently, channels in steady state characterized by uniform uplift rates and bedrock erodibility should show constant *k*_sn_ values along their course. Variations in the *k*_sn_ values can be interpreted in terms of (a) erodibility contrast, (b) variable uplift rates, or (c) non–steady state drainage systems (equations (1), (2), (3), (4)) as frequently observed in midlatitude glaciated mountain ranges. Local variations along the course of a channel indicate knickpoints in the longitudinal river profiles (Figures 5 and 6). To explore potential effects of lithology on *k*_sn_ and test whether GPS‐derived uplift rates based on short time series reflect the long‐term uplift history, we have superimposed the ESA drainage system color coded for *k*_sn_ on a map showing the lithology (Figure 5a) and plotted *k*_sn_ against uplift rate (Figures 5b–5d).

Overall, streams of the ESA feature strong variations in their *k*_sn_ values. Highest *k*_sn_ values occur in the Adige catchment at the northwestern indenter tip and at the westernmost tributaries of the Adige River and decline toward east and south (Figures 5a, 5c, and 5d). The decrease in *k*_sn_ follows both a transition from rocks of the Southalpine basement (metamorphic, plutonic, and volcanic rocks) to the cover units (e.g., carbonates) and a decrease in uplift rate (Figures 5a and 5b). We further observe elevated *k*_sn_ values in channels north of the Valsugana thrust and Fella fault compared to channels located south of these major tectonic structures. Local variations in *k*_sn_ are frequently observed in the western catchments (i.e., Adige and Brenta) and are less frequently observed in the eastern catchments (i.e., Piave and Tagliamento) and thus also show a northwest‐southeast trend. The Adige main channel is characterized by very low *k*_sn_ values with some high *k*_sn_ segments between the headwaters and the city of Meran/Merano. Downstream of Meran/Merano the channel gradient and thus *k*_sn_ values of the Adige main channel are very low compared to the *k*_sn_ values of adjacent tributaries. These low *k*_sn_ values spatially correspond to the occurrence of unconsolidated Quaternary sediments forming the river bed indicating that alluviation has been the dominant fluvial process since the glacial retreat (Figures 5a and 6b). At the confluence of the Adige River and the Torrente Avisio, *k*_sn_ values significantly increase (*k*_sn_ > 200 m) along a short channel segment upstream of Trento. Some tens of kilometers south of Trento, a channel segment of increased *k*_sn_ values tends toward the city of Verona. Compared to the receiving Adige River, the *k*_sn_ values of its largest tributary, the Eisack River, are increased by several orders of magnitude near their confluence. The same peculiarity is also observed at the confluences of the Adige River with the nearby Talferbach (Torrente Talvera), the Torrente Avisio, and several smaller tributaries. Clearly, the highest *k*_sn_ values coincide with the occurrence of metamorphic and volcanic rocks from the Southalpine basement (Figure 5a). However, these regions are also characterized by the highest amount of exhumation, implying significant long‐term surface uplift, which also affects channel gradients, and thus the *k*_sn_ values. The Adige catchment north of Trento is characterized by the highest GPS‐derived uplift rates, exceeding 2.5 mm yr^−1^ in the Ortler massif west of the Giudicarie fault and highest *k*_sn_ values of the study region (Figures 4c and 5a).

The Brenta River shows very low *k*_sn_ values in the upper reach draining the Sugana valley confined by the Belluno thrust and the Valsugana thrust in the south and the north, respectively (Figure 5a). This low *k*_sn_ section shows a prominent wind gap (Figure 5a) at the westernmost termination of the Sugana valley to about 300 m lower lying Adige valley. After leaving the Sugana valley, the *k*_sn_ values increase significantly and remain fairly constant downstream to the foreland basin. Tributaries of the Brenta catchment feature very high *k*_sn_ values north of the Valsugana thrust, where rocks of the Southalpine basement are exposed and mostly Late Miocene fission track and (U‐Th‐Sm)/He ages from apatite were found [*Zattin et al*., 2003; *Heberer et al*., 2016]. High *k*_sn_ values are also determined in tributaries draining carbonatic units featuring plateaus with reduced surface runoff due to extensive karstification (e.g., the Asiago plateau). In the Brenta catchment, high‐uplift rates are coincident with high *k*_sn_ values. This trend is best observed at the tributaries north of the main stream (north of the Valsugana thrust) but perturbed by a series of knickpoints. While we did not observe a coincidence between uplift rate and *k*_sn_ in the low‐gradient Sugana valley (upper reach of the Brenta), the lower reach of the Brenta features *k*_sn_ values that roughly reflect the GPS‐measured vertical velocity field.

The Piave catchment is characterized by fairly uniform *k*_sn_ values south of the Valsugana thrust and larger fluctuations north of it (Figure 5a). Carbonates of the Southalpine cover units are prevailing, whereas clastic rocks are exposed in the Belluno basin and at rising anticlines near the Montello‐Conegliano thrust. The Piave River crosses several major thrust faults and has been deflected due to growing anticlines at these tectonically active structures (e.g., Montello‐Conegliano thrust and Bassano‐Valdobbiadene thrust). However, *k*_sn_ values at these river sections do not indicate a significant impact of these faults on the drainage system. Distinct deviations in *k*_sn_ values occur at the headwaters of the Piave River where the channel follows the Valsugana thrust and intersects with the Fella fault. Tributaries north of the Valsugana thrust show higher *k*_sn_ values than the Piave main channel but feature some fluctuations in *k*_sn_ along their course indicating knickpoints.

The Tagliamento drainage system shows low variations in *k*_sn_ and mainly a uniform lithology consisting of carbonates from the Southalpine cover units (Figure 5a). Deviations in the main channel toward lower *k*_sn_ values are observed between the Maniago and the Arba‐Ragogna thrust, where the course of the Tagliamento River appears to be redirected due to a rising anticline, and in the orogen‐parallel channel segment east of Tolmezzo. Increased *k*_sn_ values occur in tributaries north of the Fella fault where exhumation has exposed rocks from the Southalpine basement units. Overall, south directed channel segments feature higher *k*_sn_ values than orogen‐parallel segments that follow large tectonic lineaments. The latter is subjected to river piracy as reported for the easternmost tributary of the Tagliamento River, the Fella River, that drained eastward in pre‐Pliocene times [*Monegato and Stefani*, 2010; *Monegato et al*., 2010; *Monegato and Vezzoli*, 2011]. Clearly, river piracy events suddenly increase the contributing drainage area of the “aggressor river” as manifested in higher *k*_sn_ values downstream of the capture point. Uplift rates exceeding 1 mm yr^−1^ are reported for the headwaters north of the Fella fault and lower values in the southern part of the catchment with values close to 0 mm yr^−1^. In the Tagliamento catchment, the highest uplift rates and *k*_sn_ values are spatially coincident and a low *k*_sn_ segment of the Tagliamento River near Tolmezzo matches the recorded subsidence by the nearby GPS station [*Serpelloni et al*., 2013]. Overall, *k*_sn_, lithology, and measured uplift rates match well in the streams of the Tagliamento catchment.

### 5.2 Longitudinal Channel Profiles of Major Drainage Systems

To investigate prominent features of the main longitudinal river profiles, we linked variations in *k*_sn_ to knickpoints, low‐gradient channel sections and downstream channel steepening. Similar to the *k*_sn_ analysis, longitudinal channel profiles and channel metrics derived from four major Southalpine catchments show a clear west‐east trend from distorted drainages with many knickpoints and low‐gradient channel sections in the Adige drainage system to fairly well graded channel profiles in the Tagliamento catchment. Bedrock channels are mostly observed in the steeper parts of the upper reaches, while alluvial cover prevails in lower reaches and low‐gradient channel sections. Observed knickpoints do not show a clear relation to changes in lithology (Figures 5a and 6). Number and magnitude of distinct knickpoints decrease from the Adige catchment in the west to the Tagliamento catchment in the east (Figure 6). Within the individual catchments, knickpoints in the receiving stream and major tributaries occur at similar vertical positions and show similar magnitudes. This is best observed in the Adige catchment where prominent knickpoints occur in the Adige River and the Eisack River at altitude levels of 300–500 m, 600–900 m, and 1400–1500 m (Figure 6b). Streams of the Brenta catchment feature some knickpoints between 400 m and 700 m (Figure 6c). The Piave and Tagliamento catchments feature only a few small knickpoints at an elevation range between 500 and 700 m (Figures 6d and 6e).

### 5.3 χ‐Transformed Channel Profiles

**Figure 7:**
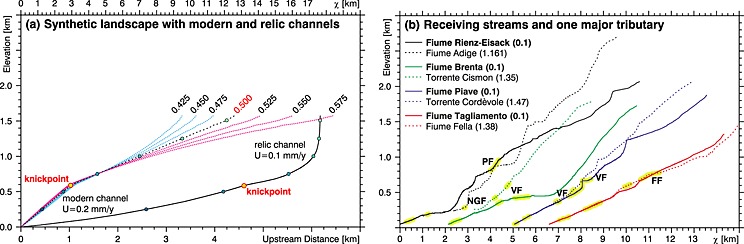
Upstream and *χ*‐transformed channel profiles. (a) Upstream channel profile (black solid line) of a synthetic perfectly fluvial but transient landscape that is described by a detachment‐limited stream power approach assuming *θ* = 0.5 and *n* = 1 (OpenLEM: S. Hergarten, personal communication, 2016). The channel profile is transformed to the χ space for *θ* (annotated) ranging from 0.425 to 0.575 (magenta dotted line: *θ*  < 0.5, black dotted line: *θ* = 0.5, and blue dotted line: *θ* > 0.5). To highlight the effect of the *χ* transformation, elevation steps of 250 m are marked by blue circles. The yellow circles with red outline indicate the position of a migrating knickpoint separating the relic channel characterized by an uplift rate of *U* = 0.1 mm yr^−1^ from the modern channel that has already adjusted to a recent uplift pulse (*U* = 0.2 mm yr^−1^). (b) Comparison of normalized *χ*‐transformed channel profiles for *θ*_ref_ = 0.5 of the receiving streams (solid lines) and one major tributary (dotted lines) of the Adige (black), Brenta (green), Piave (blue), and Tagliamento (red) catchment. Channels segments following or crossing major fault zones (Figure 6a) are highlighted by yellow stripes. NGF, North Giudicarie fault; PF, Periadriatic fault: FF, Fella fault; VF, Valsugana fault. To avoid crossing profile lines, we have shifted the profiles along the *χ* axis by adding a constant *χ* value of 0, 2, 5, and 6.5 to the profiles extracted from the Adige, Brenta, Piave, and Tagliamento catchments, respectively.

To highlight spatial variations in uplift rate and lithology and to identify temporal effects disturbing the 3D geometry of major Southalpine drainages, we employed the *χ* transformation to remove the curvature from longitudinal channel profiles (Figures 6 and 7). Before we apply the *χ* method to natural rivers, we show characteristic features of a *χ*‐transformed channel profile extracted from a synthetic alpine landscape. The channel profile features a migrating knickpoint separating an equilibrated and a relic channel segment that indicates a temporal variation in uplift rate (Figure 7a). After the *χ* transform, an equilibrium channel profile characterized by uniform uplift rates and lithology is represented by a straight line. A migrating knickpoint that indicates the adjustment of the channel toward new uplift rates is characterized by a prominent kink in the *χ*‐transformed channel profile that separates two straight channel segments with different slopes. The *χ* transform may be biased by employing incorrect *θ* values that cause a concave (too small *θ*) or convex (too large *θ*) deviation from a straight line in the *χ* space. Several methods to determine the best fit *θ* using the *χ* transform are reported in the literature [*Perron and Royden*, 2013; *Mudd et al*., 2014; *Hergarten et al*., 2016]. However, as we intend to compare *χ* and the slope of channel profiles in the *χ* space for rivers draining different Southalpine catchments, we define *θ*_ref_ = 0.5 and compute in analogy to the *k*_sn_ the *χ*_n_ (normalized *χ*) for the receiving stream and one major tributary of each investigated catchment.

The comparison of major streams of the Southern Alps in *χ* space reveals a clear trend of decreasing average slopes from the west to the east, with lowest average gradients in the easternmost Tagliamento catchment (Figure 7b). This trend is consistent with decreasing *k*_sn_ from west to east (Figure 5) and goes along with increasing linearity of *χ*‐transformed profiles, from the highly distorted Adige catchment to the fairly well equilibrated Tagliamento catchment. The Rienz‐Eisack River deviates from this trend with an upstream decreasing slope of its *χ*‐transformed profile but features also a conspicuous T‐shaped river junction and a prominent wind gap at the Adige‐Drau drainage divide (Figure 1). The Brenta main trunk is characterized by a low‐gradient section draining the Sugana valley but shows the described west‐east steepening trend in the upper reach north of the Valsugana Thrust.

All major rivers feature a considerable linear channel segment at the lower reaches and steeper but more distorted segments in the headwaters. Pronounced low‐gradient segments occur in the two western catchments but are missing in the eastern drainages. Interestingly, the steepest segments in *χ* space occur north of the Valsugana‐Fella fault system that corresponds to the highest measured uplift rates.

## 6 Discussion

A detailed analysis of major streams and tributaries of the Southern Alps revealed a series of conspicuous morphological features in alpine valleys such as the spatial trends in the *k*_sn_ pattern, knickpoints, low‐gradient channel sections, and asymmetric drainage patterns. We discuss these characteristics in terms of contrasting bedrock lithology, spatial variations in uplift rate, and base level changes as a consequence of the Pleistocene glaciations and the formation of anticlines by the late initiation of uplift along the frontal thrusts. We further discuss Alpine topography formation in the light of base level changes in the far field of the Alps such as the desiccation of the Mediterranean Sea and the inversion of the northern Molasse Basin.

### 6.1 Effects of Bedrock Lithology on Channel Metrics

The influence of bedrock lithology on *k*_sn_ (Figure 4b) is evidenced by numerous studies [e.g., *Duvall et al*., 2004] and also observed in the ESA (Figure 5a). There, *k*_sn_ deviates significantly from the simplified assumption of steady state channels with uniform *k*_sn_ draining a region characterized by uniform uplift rates and lithology. The ESA display a trend of higher *k*_sn_ values in the northwestern and lower *k*_sn_ values in the southeastern part of the study region. This trend corresponds roughly with the overall amount of exhumation. Metamorphic and volcanic rocks from the Southalpine basement are partly exposed in the Adige and Brenta catchments, while lithology is less heterogeneous and mostly restricted to carbonates of the sedimentary cover in the Piave and Tagliamento catchments. In addition, some major jumps in *k*_sn_ along streams coincide with changes of the bedrock lithology as observed in tributaries of the Adige (e.g., Eisack River, Torrente Avisio, and Talferbach) and the Brenta Rivers (e.g., Torrente Cismon, Torrente Maso) (Figures 5a and 6). This suggests that some bedrock types of the Southalpine basement (e.g., Bozen Quartz Porphyry) are characterized by a low erodibility and thus may be responsible for steeper channels (Figures 4b and 5a).

### 6.2 Long‐Term Exhumation, Present Uplift Rates, and Long‐Term River Adjustment

Long‐term exhumation in the ESA has clearly focused along thrust faults, as indicated by the exposure of crystalline basement along, e.g., the Valsugana thrust and as quantified by low‐temperature thermochronology data [e.g., *Zattin et al*., 2006; *Heberer et al*., 2016] (see chapter 2.3 for details). Pronounced and persistent gradients in the vertical and horizontal velocity field have led to the largest overall exhumation in the upper Adige catchment as well as north of the Valsugana‐Fella fault system in the upper reaches of the Brenta, Piave and Tagliamento catchments (Figures 4d and 5a). GPS‐based uplift rates ≥ 2 mm yr^−1^ in the northern and western part of the ESA exceed reported exhumation rates by about 1 order of magnitude. Nonetheless, we observe a decrease of exhumation level from the northwest to the southeast, which is in line with the GPS‐derived uplift pattern (Figure 4c) and is also reflected in *k*_sn_ (Figure 5). The clear relation between uplift rate, exhumation pattern, and *k*_sn_ in the ESA shows (a) that the GPS‐derived, short‐term uplift pattern is consistent with long‐term topography formation (despite a difference in absolute rates), and (b) that the drainage system generally reflects variations in uplift and lithology (Figures 4b and 5) despite the impact of the Pleistocene glaciations on the geometry of major valleys (Figure 4f). However, large fluctuations in channel slope may reflect the glacial impact on ESA channels. *Robl et al*. [2015] showed that the standard deviation in slope strongly increases with the occurrence of glacial landforms and reaches a maximum above the LGM equilibrium line altitude.

Additional evidence for the link between uplift rate and channel geometry can be found in *χ*‐transformed channel profiles (Figure 7b). Here we observed a clear east to west increase in slope from the Tagliamento River featuring uniform but only low positive uplift rates to the headwater of the Adige River characterized by high positive uplift rates (Figure 7b). This observation may be amplified by contrasting bedrock erodibility. However, the bedrock of the Piave River and the Tagliamento River is composed of carbonates from the sedimentary cover (Figure 5a), allowing a direct comparison of these rivers and their tributaries. The Piave is affected by higher uplift rates and consequently shows a steeper *χ*‐transformed channel profile (Figure 7b) and increased *k*_sn_ values in most tributaries (Figure 5a).

GPS‐derived uplift rates exceeding 1 mm yr^−1^ are mainly observed north of the Valsugana‐Fella fault system. This important tectonic structure also marks a distinct discontinuity in channel metrics by separating low‐ and high gradient segments of *χ*‐transformed channel profiles (Figure 7b) and by a significant increase in *k*_sn_ (Figure 5a) north of this major fault zone. This evidences that crustal shortening due to indenter tectonics has a significant impact on channel metrics in the ESA.

However, the locus of highest uplift affecting ESA and EA domains in the northwestern part of the Adige catchment, and an eastward decrease in uplift rate, can hardly be reconciled with counterclockwise rotation of the Adriatic plate around an Euler pole near Milano, which has to result in an eastward increase in convergence rate, crustal shortening, and consequently uplift (Figure 1). This demands for an additional uplift mechanism and questions the dominant role of the observed indentation of the Adriatic plate in controlling topography formation in the northwestern part of the ESA. Interestingly, the area of highest uplift rates at the NW tip of the indenter is located above a slab gap at depth and a controversially discussed switch in subduction polarity as revealed by seismic tomography [*Lippitsch et al*., 2003; *Kissling et al*., 2006; *Mitterbauer et al*., 2011; *Handy et al*., 2015]. Alternative sources of uplift such as slab break‐off, Late Miocene stress field inversion and isostatic response to erosional unloading during glacial‐interglacial periods have been proposed for the European Alps and may contribute to the observed uplift pattern [e.g., *Horváth et al*., 2006; *Champagnac et al*., 2007; *Fox et al*., 2016]. The latter process contributes significantly to the present uplift pattern of the Western and Central Alps [*Gudmundsson*, 1994; *Champagnac et al*., 2007; *Norton et al*., 2010; *Champagnac et al*., 2012], and we expect that isostatic response may also be an important source of uplift in the ESA‐EA realm. Accumulation of ice is restricted to areas above the snowline that control the extent and duration of glacial occupation. Hence, we expect that glacial‐driven erosional unloading declined in the study region roughly from northwest to southeast during the Pleistocene glaciations. The resulting uplift pattern would be consistent with spatial gradients of GPS‐determined uplift rates and channel metrics in the ESA. Besides these general observations, deviations such as low‐gradient channel sections in high‐uplift areas and major knickpoints can be observed (e.g., Adige River) (Figure 6).

### 6.3 The Origin of Major Knickpoints

The discussed spatial variations in uplift rate and bedrock erodibility serve to explain large‐scale topographical features of the ESA, but most low‐gradient channel sections and major knickpoints are not located at distinct breaks in lithology or uplift rate and hence may indicate temporal variations of the external drivers (Figures 3, 5, and 6). In the ESA, three time‐dependent mechanisms have been reported that led to base level changes affecting the evolution of the Alpine landscape. These are (a) southward thrust propagation mainly since the Miocene [e.g., *Castellarin and Cantelli*, 2000], (b) the desiccation of the Mediterranean during the Messinian salinity crisis [e.g., *Willett et al*., 2006], and (c) glacial carving during the Middle and Late Pleistocene [e.g., *Muttoni et al*., 2003].

The magnitude of the Messinian incision in the ESA may vary between individual catchments with respect to the bathymetry of the desiccated Adriatic basin, which was divided by a flexural bulge at that time [*Mancin et al*., 2009]. Incision in the Venetian Plain did not exceed 300 m [*Mancin et al*., 2009], and we expect this to be a good proxy for bedrock incision of the Tagliamento River and Piave River. However, the Adige catchment further west may have experienced a larger base level drop and thus more intense incision. If we take the recent vertical velocity field with uplift rates ranging mostly between 1 and 2 mm/yr in the Adige and 0.1 and 0.5 mm/yr in the Tagliamento catchment (Figure 4d) as a proxy for the last few million years, Messinian knickpoints should have been uplifted by about 5–10 km in the upper Adige catchment and more than 0.5–2.5 km in the Tagliamento catchment. This would imply that propagating Messinian knickpoints have already left at least the drainage system of the high‐uplift Adige catchment (Figure 4d). Such theoretical scenario would have clearly left its imprint on the thermochronological pattern, which is, however, not the case: Neither apatite fission track and (U‐Th)/He data from the Adamello batholith [*Reverman et al*., 2012] nor from the Giudicarie belt and adjacent regions [*Heberer et al*., 2016] document an increase in exhumation rates related to the Messinian salinity crisis.

The Messinian base level drop is evidenced by relics of deeply incised channels buried by sediments and observed below major lakes (e.g., Lake Garda) [e.g., *Preusser et al*., 2010] and at river mouths of alpine streams, which is consistent with subsidence indicated by the GPS‐derived uplift pattern for these domains (Figure 3c). The observation that the Messinian incision wave has never reached the high alpine domains in an intensity to be detected by low‐temperature thermochronology demands for either the decay of knickpoints during upstream migration [*Royden and Perron*, 2013] or the (partial) burial of knickpoints beneath sediments deposited during the post‐Messinian sea level rise preventing further upstream migration (compare temporal evolution of the southern channel in Figure 4d). The latter implies that steep Messinian channels got stuck in valley segments that have not been significantly uplifted since Messinian times as observed at the transition from the orogen to the foreland in the ESA [*Mancin et al*., 2009; *Preusser et al*., 2010] and as reported for the Rhone catchment draining a major part of the Western Alps [*Loget et al*., 2006]. A partial burial of knickpoints is conceivable where only a fraction of the oversteepened Messinian channels migrated upstream. These knickpoints might still be visible in low‐uplift areas such as the Tagliamento catchment (Figure 6e). In such a scenario, the magnitude of exhumation would fall below the limit of detection by methods of low‐temperature thermochronology.

Major knickpoints in channels of the European Alps originated after the Middle Pleistocene climate transition by the development of characteristic glacial landforms such as overdeepened and hanging valleys [e.g., *Robl et al*., 2008a; *Valla et al*., 2010]. We suppose that prominent knickpoints observed in the ESA are also glacially induced. In the Adige catchment, we observe three (maybe four) distinct knickpoints in the Adige River at elevation levels of about 500, 900, 1400 m, which are paralleled by knickpoints at similar elevations in the Eisack River. We propose that each knickpoint pair has a common origin downstream the confluence of the Adige River and the Eisack River (Figures 6a and 6b) from where they have migrated upstream the drainage system driven by both glacial [e.g., *Shuster et al*., 2011] and fluvial [e.g., *Wobus et al*., 2006] processes (Figure 4f). In such a case, knickpoints at different levels would represent the temporal succession of glacial advances during the Pleistocene glacial cycles. This is supported by the absolute vertical position of the highest knickpoints and the drop between the different knickpoint levels, which are consistent with (a) an uplift rate of about 1–2 mm yr^−1^ as determined by *Serpelloni et al*. [2013] for the upper Adige catchment since the onset (~0.9 Ma [*Muttoni et al*., 2003]) and (b) the cyclicity of the Pleistocene glaciations, respectively. These assumptions roughly constrain the vertical knickpoint migration rate to about 1–2 mm yr^−1^.

Furthermore, buried Messinian knickpoints could be partially revived by Pleistocene glacial sediment excavation. We speculate that glaciers overdeepened alpine valleys preconditioned by Messinian incision, and that major knickpoints observed in streams of the Adige catchment originate from relic Messinian channels exposed by repeated glacial erosion. The excavation of rocks during the Pleistocene glaciations is most spectacularly observed at the overdeepening of Lake Garda but is also consistent with the low lying and more or less flat valley floor of the Adige River downstream of Meran/Merano.

The Brenta River shows a low‐gradient section at the Sugana valley confined by a distinct knickpoint followed by downstream steepening (Figures 5, 6, 7). This channel morphology is most likely attributed to the time lag between river adjustment and the uplift of the Sugana valley, but the data sets presented do not suffice to infer cause and timing of the associated base level change. The Piave River flows through the Belluno Basin representing a similar tectono‐morphological feature as the Sugana valley (Figures 5, 6, 7). In contrast to the Brenta River, the Piave River features a well‐graded channel profile and uniform *k*_sn_ values at this section, although the main channel of the Piave River was deflected in post‐Messinian times by the growing Monte Grappa anticline (uplifting also the Belluno Basin). We suggest that the adjustment of the Piave River to uplift and deflection is already accomplished in this channel segment due to its larger contributing drainage area (and hence a higher discharge) compared to the Brenta River. We further suggest that deflection might be unlikely for the Piave River featuring high discharge in a steep, deeply incised channel as proposed for post‐Messinian times [*Monegato et al*., 2010] and suggest that Messinian channels may have already been filled with sediments at the onset of anticline formation. However, the question remains how the increase in flow length of the main channel has affected the drainage network topology and the evolution of topography in the headwaters of the Piave River. In principle, this should lead to an increase in mean elevation of the catchments north of the Belluno basin and to a loss of catchment area to adjacent streams not affected by the deflection. We hypothesize that the low number of tributaries east of the main channel may be the consequence of channel deflection triggering drainage network reorganization. We wonder whether the similar vertical positions of knickpoints in the catchments of the Piave River and the Tagliamento River may be attributed to the Messinian signal reactivated by glacial erosion tantamount to those discussed for the Adige River.

### 6.4 Constraints on the Stability of Drainage Divides

**Figure 8:**
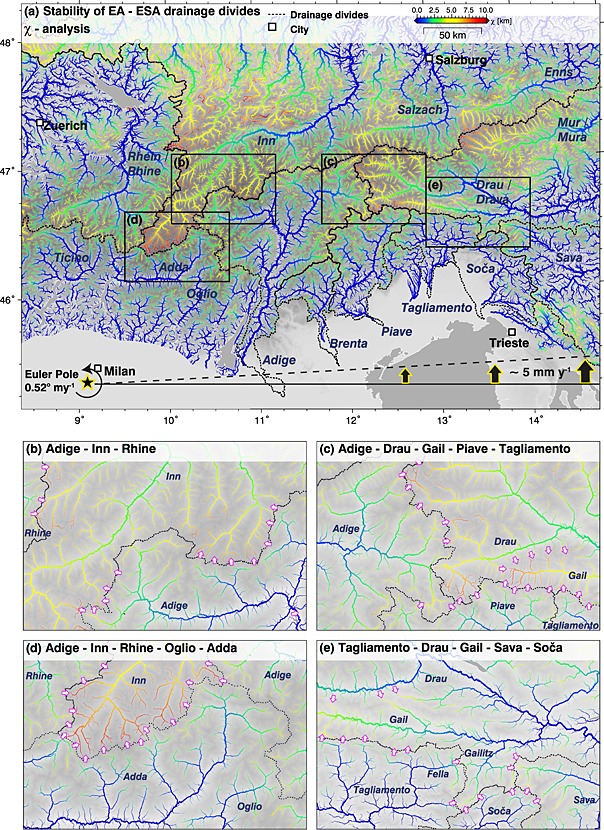
(a) Spatial distribution of *χ* in channels of the Southern and adjacent Eastern Alps for *θ* = 0.5 and a common base level of 500 m where *χ* = 0. Note that ESA channels below 500 m are abundantly characterized by alluviation and a transport‐limited regime and that a lower base level (e.g., 300 m) results in larger gradients in *χ* across ESA‐EA watersheds. Channels with *A* > 1 km^2^ are plotted, and the width of channels is proportional to log_10_(*A*). Purple arrows indicate the expected direction of drainage divide migration. Important rivers are annotated. (b–e) Key areas are enlarged and their extent is indicated by rectangle in Figure 8a.

Migration of watersheds and stream captures are documented in the ESA. For example, the orogen‐parallel channel segment of the Fella River, a former tributary of the Sava River, was captured by the Tagliamento River in post‐Miocene times (Figure 5). This event resulted from a north directed shift of the Tagliamento drainage divide [*Monegato and Stefani*, 2010; *Monegato et al*., 2010]. Figure 8 shows *χ* maps for drainages of the Southern and adjacent Eastern Alps. It is easily recognized from equation (9) that regions with low actual erosion rates *E*, e.g., due to low‐uplift rates, should feature high *χ* values. If such regions are far away from the considered drainage divide, the resulting high *χ* values may be misinterpreted in terms of migrating drainage divides. A strong increase in *χ* is observed in the orogen‐parallel, low‐gradient valleys of the EA with valley floors located at an elevation roughly between 400 and 500 m. These valleys are filled by thick layers of Miocene to Quaternary sediments [e.g., *Preusser et al*., 2010] indicating that rivers in these segments are transport limited and depositing sediments rather than incising into the bedrock. In order to avoid misinterpretation regarding the mobility of watersheds in the EA‐ESA realm caused by alluviation in orogen‐parallel valleys, we defined a base level of 500 m in the *χ* maps. We note that by defining base levels below 500 m and considering all orogen‐parallel river segments of the EA, the *χ* pattern is qualitatively the same but the differences in *χ* across the EA‐ESA watersheds increase dramatically.

The *χ* map for drainages of the Southern and adjacent Eastern Alps computed for a 500 m base level is consistent with the observed trend of north directed drainage divide migration [*Monegato and Stefani*, 2010; *Monegato et al*., 2010] (Figure 8). While there are small gradients in *χ* across watersheds of the ESA rivers, large gradients still occur across watersheds separating streams from the ESA (Adige River, Piave River, and Tagliamento River) and the EA (Inn River, Gail River, and Drau River). The *χ* map implies that the westernmost branch of the Adige headwaters is expected to grow toward the west and north by capturing tributaries of the Inn catchment (Figures 8a, 8b, and 8d). Further east, the Adige, Piave, and Tagliamento drainage systems may grow at the expense of the Gail and Drau drainage systems by divide migration and river piracy (Figures 8a, 8c, and 8e). A strong gradient in *χ* across the Adda‐Inn watershed (Figure 8d) west of the Adige catchment and a similar situation at the Soča‐Sava drainage divide (Figure 8e) east of the Tagliamento catchment indicate a north directed, orogen‐scale drainage divide migration.

In principle, gradients in *χ* across drainage divides as observed in the ESA‐EA domain may also result from spatial variation in uplift rate or even bedrock erodibility; hence, stable watersheds may be erroneously interpreted as migrating [*Willett et al*., 2014]. Indeed, we observe an increase in uplift rate (consistent with *k*_sn_) from the southern foreland toward the ESA‐EA drainage divide. However, GPS‐based uplift rates [*Serpelloni et al*., 2013] indicate a similar trend for the EA rivers with highest uplift rates near the ESA‐EA watershed. Evidence that the uplift rates in the ESA are higher than in the EA is missing. Figure 8a indicates that the Inn River shows significantly higher *χ* values than the Rhine River in the west, the Adda River in the south, and the Adige River in the east. Interpreting this *χ* pattern in term of spatial variations in uplift rate would mean that the Inn catchment occupies a low‐uplift corridor, surrounded by catchments draining higher uplift areas, which seems very unlikely and is not supported by the present vertical velocity field. Furthermore, it is common knowledge that the Rhine catchment is growing at the expense of the Danube catchment [e.g., *Berendsen and Stouthamer*, 2001; *Kuhlemann and Rahn*, 2013]. Together with strong evidence from the Southern Alps [*Monegato et al*., 2010], these field observations strengthen our interpretation of the *χ* maps in terms of a north directed migration of ESA‐EA watersheds.

Several counteracting processes may be responsible for an expected shift of the drainage divide separating the EA from the ESA (Figure 4).

1. Shortening of the Adriatic indenter accompanied by the southward propagation of thrusts induces surface uplift and controls the lateral growth of the Southern Alps [e.g., *Castellarin and Cantelli*, 2000; *Zattin et al*., 2006]. This should lead to a south directed shift of the watershed between the ESA and the EA (Figure 4c).
2. The Messinian incision wave triggered by the desiccation of the Mediterranean Sea exclusively affected drainages of the ESA [e.g., *Willett et al*., 2006] and should result in a north directed shift of EA‐ESA drainage divide followed by a backward migration after the incision wave has left the system (Figure 4d).
3. The EA experienced Late Miocene relief rejuvenation [*Hergarten et al*., 2010; *Legrain et al*., 2014a; *Robl et al*., 2015] accompanied by a significant uplift of the former stable base level of rivers north of the EA‐ESA drainage divide, the northern Molasse, and Pannonian basins [*Genser et al*., 2007; *Gusterhuber et al*., 2012; *Baran et al*., 2014]. This process should result in a north directed shift of the EA‐ESA drainage divide (Figure 4e).
4. Localized erosion during the Pleistocene glaciations may have affected the state of drainage divides as well (Figure 4f), but we expect that glacial perturbation and the resulting drainage divide reorganization are spatially limited compared to the processes described above.

The observed and expected northward migration likely reflects base level changes in the far field, rather than uplift driven by indentation or the effect of Pleistocene glaciations. In the case of (2), the shift of the divide would be reversible and should affect the entire ESA‐EA watershed. In contrast, (3) can be expected to lead to a permanent north directed migration of the watershed which should be restricted to the ESA‐Danube drainage divide. Spectacularly, *χ* maps indicate that the Adda‐Rhine watershed is stable with similar *χ* on both sides of the watershed, while the Rhine catchment grows at the expense of the Danube drainage system (Inn catchment) (Figures 8a, 8b, and 8d). This supports scenario (3), an inversion of the northern foreland basin, possibly in concert with dynamic reorganization of the Rhine drainage system [*Kuhlemann and Rahn*, 2013].

The observations reported above imply that the Periadriatic fault system no longer represents a strong tectonic discontinuity with respect to the present and future evolution of drainage systems. Thus, the drainage divide tracing the Adriatic indenter may vanish as watersheds rearrange according to the long‐term vertical velocity field and base level changes in the far field.

## 7 Conclusions

We analyzed the topography of the eastern Southern Alps (ESA) and adjacent domains of the Eastern Alps (EA) based on drainage pattern indices and their relation to regional tectonic and climatic characteristics and found strong evidence for a northward shift of the ESA‐EA drainage divide. We conclude the following.

1. Fault zones control the flow direction of major rivers in the ESA, deform the drainage pattern, and advect streams horizontally and vertically.
2. Modern uplift rates are largely consistent with long‐term exhumation in the ESA and with variations in normalized steepness index (*k*_sn_) indicating a stable uplift and erosion pattern since Miocene times. Uplift rates and *k*_sn_ decrease from the indenter tip in the northwest toward the southeast, which is probably not a result of crustal shortening due to the kinematics of indentation alone.
3. The Valsugana‐Fella fault system marks a distinct morphological discontinuity with an increase in channel steepness north of this tectonic structure, which is consistent with shortening and crustal thickening due to indentation. Variations in rock type may have amplified the observed *k*_sn_ pattern as rocks from the Southalpine basement and cover units outcrop in high‐ and low‐uplift areas, respectively.
4. Major knickpoints of the Adige River, the Piave River, and the Tagliamento River cluster at similar vertical positions, indicating a common origin within the individual catchments. Most knickpoints probably evolved during Pleistocene glaciation cycles but may represent the incrementally reactivated, buried incision signal triggered by the Messinian desiccation of the Mediterranean Sea.
5. The EA‐ESA drainage divide separates high *χ* drainages of the EA from low *χ* drainages of the ESA. This implies that ESA streams spread to the domain of the EA by drainage divide migration and river capture events. As already observed in the Adige catchment, the Periadriatic fault system loses its significance for the morphological evolution of the EA‐ESA.
6. The observed northward migration of the ESA‐EA drainage divide is most likely driven by a base level rise in the northern Molasse basin, which leads to a growth of the ESA and Rhine catchments at the expense of the Danube drainage area.

This study contributes to an enhanced understanding of the complex topographic evolution of the ESA controlled by the interplay of continental collision and indenter tectonics, Late Miocene base level changes, and Pleistocene glaciations. Future research should focus on constraining the pace and the direction of drainage divide migration in the entire European Alps to further disentangle processes and drivers of alpine landscape evolution.
